# Information-seeking behaviour in patients exploring orthognathic surgery: A qualitative study

**DOI:** 10.1177/14653125241249494

**Published:** 2024-05-08

**Authors:** Nicola Wade, Ninu Paul, Nathan Nagar, Sarah Rolland, Sarah Germain

**Affiliations:** 1 Orthodontic Department, Cumberland Infirmary, Carlisle, UK; 2 School of Dental Sciences, Newcastle University, Newcastle upon Tyne, UK

**Keywords:** consent, decision-making, patient information, orthognathic

## Abstract

**Objective:**

To explore how orthognathic patients seek information during decision-making.

**Design:**

Qualitative, cross-sectional study.

**Setting:**

A hospital in Cumbria, UK.

**Participants:**

Prospective orthognathic patients.

**Methods:**

Participants were purposively recruited from joint orthognathic clinics after the original consultation. Semi-structured interviews were conducted via remote video call with nine participants aged 18–30 years. Data collection and reflexive thematic analysis occurred in parallel until thematic saturation was achieved.

**Results:**

The central finding of this research was that patients were making informed decisions about orthognathic surgery. Four themes were identified to support this central finding including the following: (1) selective engagement with orthognathic information sources; (2) the central role of patient-specific information from professionals and peers; (3) Internet use to supplement standard information resources; and (4) concerns over information found online. The preferred source of information was verbal from the clinical team as it was trusted and person-specific. Past patients were identified as valued sources of information and establishing contact through digital social media networks was found to be a convenient alternative to face-to-face. Online information found was valued but concerns included information overload, problems establishing applicability and concerns over its credibility.

**Conclusion:**

Orthognathic patients were making informed decisions about their treatment. This study highlights the central role of the patient–clinician interaction in decision-making, especially in providing patient-specific information. Insight into the nuances of information-seeking behaviours will better inform clinical care. Since patients frequently access online information that is decision-relevant, encouraging patients to discuss online searches will support the shared decision-making process and alleviate any concerns with information found. During consultation, explaining the purpose of an information aid rather than expecting patients to read them separately, may further enhance its usefulness in decision-making. This study identified an unmet need for visual aids, such as real-time images of postoperative recovery. These findings can inform the design of future information resources.

## Introduction

Orthognathic treatment is a major elective procedure offered to patients with significant dentoskeletal discrepancies. It generally involves approximately three years of treatment, surgical risks and results in noticeable facial changes.

Information and shared decision-making in orthognathic care is an ethical and legal requirement and leads to greater patient satisfaction (Chen et al., 2002). A wide range of information sources are available to orthognathic patients. These resources should meet the identified needs of the patients in order to best support decision-making. Current research involving the assessment of information in this field has been from a clinician’s perspective, evaluating what professionals believe patients should be told, rather than identifying what information patients value and in what form (Aldairy et al., 2012; Hegarty et al., 2017; Seehra et al., 2016). Most of the literature from a patient’s perspective is derived from patient satisfaction questionnaires, which have related postoperative dissatisfaction to a lack of information (Al-Hadi et al., 2019; AlKharafi et al., 2014; Cunningham et al., 1996; Finlay et al., 1995; Pacheco-Pereira et al., 2016). Although useful, quantitative studies cannot explore in depth the information-seeking behaviours of patients.

A qualitative methodology offers a means of exploring information-seeking fully. There remains a lack of qualitative research exploring orthognathic patients’ information-seeking behaviours specifically during decision-making. The majority of studies investigate decision-making as a whole, information being just one theme identified (Broder et al., 2000; Paul et al., 2022; Stirling et al., 2007). The few studies (Patel et al., 2017; Santos et al., 2012) that focus on information-seeking do so using postoperative samples and focus on information about surgical aspects of care and not all decision-relevant information. A major drawback of studying a postoperative sample, with patients undergoing treatment several years previously, is recall bias, particularly regarding the information received before committing to surgery. Therefore, there is a need to study a preoperative sample that would offer more accurate descriptions of the information used during decision-making.

The aim of the present study was to explore how patients seek information about orthognathic treatment during decision-making, identify preferred methods and perceived barriers from a preoperative patient’s perspective, through qualitative inquiry.

## Participants and methods

This was a qualitative, cross-sectional study. Ethical approval was granted from Leicester South Research Ethics Committee (Ref. 20/EM/0303). Preoperative participants were recruited from joint orthognathic clinics at North Cumbria Integrated Care NHS Foundation Trust after an initial consultation. Maximum variation purposive sampling was used to capture a wide range of perspectives. Patients aged under 18 years and patients with syndromic craniofacial or cleft lip and/or palate were excluded to reduce the complexity of ethical process and to represent the ages at which most patients are making an independent decision to undergo orthognathic surgery. Potential participants were approached on clinic by the direct care team and invited to take part in an interview. A participant information sheet, consent form and invitation letter were provided. A detailed step-by-step guide to accessing online interviews was also provided to potential participants (See Appendix A). Participants interested in taking part were contacted by the researcher (NW) to arrange an interview. Interviews only proceeded once informed, written consent was obtained. The sample size was determined by thematic saturation, the point at which little or no relevant new themes or codes are identified within the data.

Semi-structured interviews were conducted via remote video call using Microsoft Teams. The remote interviews made the study feasible during the pandemic and interviewees were alone in a non-clinical setting, at home. The interviewer (NW) was an orthodontic trainee, self-taught qualitative researcher with no direct involvement in orthognathic patient care and introduced as a researcher to create a safe space for open and candid discussion. The researcher was aware of their role and practised principles of reflexivity throughout, including constant self-appraisal and reflective notes. For example, as Braun and Clarke (2021) recommend, the researchers were aware of their position as clinicians and drew upon its influence on how the researcher interacted with the participant at the interview and also how the data were analysed. The interview and questioning styles were conducted as recommended by Britten (1995) using a topic guide. The interview started with easy questions then more probing questions asked in an open-ended, neutral and clear manner. The topic guide was produced from a literature review and further developed during the research to explore themes further (see Appendix B). The literature review was used as a guide with questions based on previous knowledge and gaps in knowledge. The probes were modified as the research progressed to explore the themes that emerged in previous interviews. The interviews were audio recorded and transcribed verbatim into Microsoft Word by a researcher (NW) with participants’ names pseudo-anonymised. No repeat interviews were undertaken. The transcripts were not sent to participants for corrections or comments. Reflective thematic analysis was conducted following guidelines outlined by Braun and Clarke (2021), a flexible approach and compatible with the constructivist stance taken. For clarity of process, Table 1 outlines the six steps used in analysis. Two researchers (NW, NN) independently coded the data. Through discussion between researchers (NW, NN and SG) an agreed set of themes was identified using an inductive approach. The themes were then critically appraised by the entire research team, which included some experienced and trained qualitative researchers (NW, NN, NP, SR, SG) to ensure accuracy and remove possible preconceptions, thus defining and naming the final themes. In addition, the rigorous process of data analysis in a phased manner added to the rigour and trustworthiness of the study. NVivo 12 software was used to assist data organisation and retrieval (NVivo, QSR Version 12, 2018). Reflective notes were made shortly after the interviews throughout the process. The study followed the standard criteria of reporting qualitative studies: COREQ checklist (Tong et al., 2007).

**Table 1.** Stages of data analysis adapted from Braun and Clarke (2006).

Stage	Description
**1.** Familiarisation with data	Data transcription (NW) and immersion by reading the data several times (NW, NN)
**2.** Identify codes	Coding by identifying a word or short phrase that represents a feature of the data that imparts one overall meaning relevant to the research question across the entire dataset, collating data relevant to each code (NW, NN)
**3.** Identify themes	Grouping codes with unifying concepts that characterise behaviours together into provisional themes (NW, NN)
**4.** Review themes	Discussion to relate themes with the literature on information seeking-behaviours and decision-making and agreeing on a common set of themes (NW, NN, SG). Finalising and reviewing themes in relation to coded extracts and the entire dataset (NW, NN, SG, SR, NP)
**5.** Define themes	Producing a name, definition, scope and exhaustive set of data to support each theme
**6.** Report	The final stage of data analysis by selecting vivid, encapsulating extracts, relating to the research question and literature and producing a report (NW, NN, SG, SR, NP)

## Results

**Figure 1.**
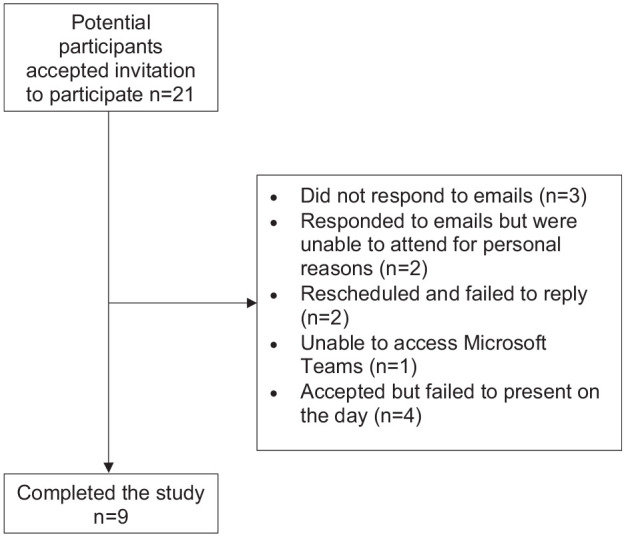
A flow chart of participant drop-out.

In total, 21 eligible participants were identified. Of the 21 approached, nine participants (four men, five women; age range = 18–30 years) were interviewed. Information about non-participation is shown in Figure 1 in the form of a flow chart. No further sampling was done since thematic saturation was attained with nine interviews (mean length = 31 min; range = 22–40 min). Information about interviewee demographics is shown in Table 2. Participants were given a unique code to describe their sex (M [male]/F [female]), a numerical identifier (superscript) and age (years). For example (F^1^ 27) describes a 27-year-old woman (identifier 1). Being an online interview, connectivity issues were experienced by one participant, but they were quickly overcome leaving no effect on the interview.

**Table 2.** Demographics of participants interviewed.

Participant	Age	Sex	Ethnicity	Malocclusion	Interview date
1	27	Female	Caucasian	Class II	02/06/2021
2	23	Female	Caucasian	Class III	03/06/2021
3	18	Female	Caucasian	Class II	10/06/2021
4	23	Female	Caucasian	Anterior Open Bite	12/06/2021
5	20	Male	Caucasian	Class III	13/07/2021
6	19	Male	Caucasian	Class II	20/07/2021
7	27	Male	Caucasian	Class III	22/07/2021
8	30	Female	Caucasian	Anterior Open Bite	11/09/2021
9	18	Male	Caucasian	Class III	09/09/2021

**Figure 2.**
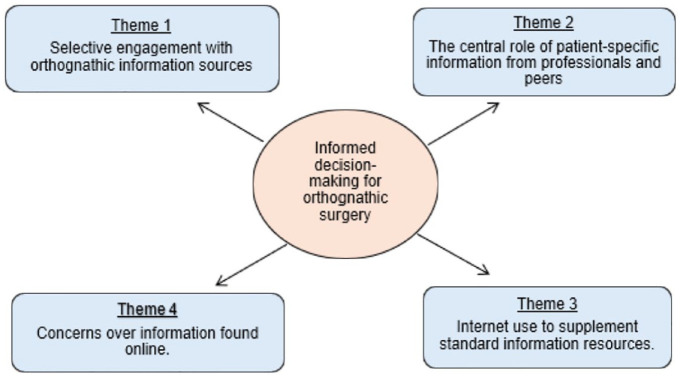
A thematic map for ‘informed decision-making for orthognathic surgery’ analysis.

The central finding of this research was that patients were making informed decisions about orthognathic surgery. Four themes (See Table 3) were identified to support this central finding including the following: (1) selective engagement with orthognathic information sources; (2) the central role of patient-specific information from professionals and peers; (3) Internet use to supplement standard information resources; and (4) concerns over information found online. For clarity, a thematic map of analysis is presented in Figure 2. Each will be presented and explored with representative quotes from the interviews.

**Table 3.** Information-seeking behaviours themes and sub-themes.

Theme	Sub-theme
Selective engagement with orthognathic information sources	Extractive approach by asking questions
	Avoidance of certain aspects of information by confirmation bias / by delegation to healthcare professional
	Reactive approach after a significant event: third molar extraction or seeing another patient in postoperative recovery
The central role of patient-specific information from professionals and peers	Participants preferred face-to-face verbal information from the clinician as it was patient-specific
	Patients valued information from past patients of the same age / stage of treatment / with the same health provider face-to-face as well as via social media and online forums
	Negative perceptions of supplementary information aids provided without discussion with professionals
	Supplementary written leaflets found to be too general with too much text / diagrams; of treatment timeline recommended by participants
Internet use to supplement standard information resources	Internet use to supplement visual before and after photographs that were found to be missing from the standard information supplied by the clinical team
	Inadequacies in visual aids of the immediate postoperative recovery period provided by the clinical team resulted in patients resorting to online social media
	Internet use to seek clarity on medical terminology
Concerns over information found online.	Patients trusted information from professional sites but did not trust unverified sites like Wikipedia
	Participants felt postoperative photographs on Instagram could have been photoshopped
	Participants has concerns with outdated information online
	Problems establishing applicability
	Information overload

### Theme 1: Selective engagement with orthognathic information

Participants were selective about what information they sought through actively asking questions, avoiding certain aspects and choosing to seek further information after a significant event. The majority of participants sought traditional resources including formal consultation with a professional before engaging in information online and other informal sources.

‘*At first, I seek the professional advice and then go on the websites that they say and then if I want to look just more into it myself, just me being more curious. I ask people that I know that are going through the same situation or look on Instagram.*’ (F^2^ 23)

These individuals adopted a shared decision-making approach and were able to engage in the process by asking questions. Some pre-prepared questions motivated by the thought of forgetting to ask certain questions:

‘*I could write a list of questions and have a proper think about it. If not, I would have run the risk of missing something, not asking something, which would have left a bit of a hole in the decision making.*’ (F^4^ 23)

Conversely, several participants avoided certain aspects of information such as the risks of surgery, accepting information that supported their decision.

‘*I was just uber positive, optimistic about the situation not willing to hear the negative side.*’ (F^4^ 23)

Trust in the clinician was another reason associated with selective information avoidance, as participants felt there was no need to seek information as the clinician was somehow more authoritative than other sources, even their own.

For some the approach changed over time, only accessing information after a significant event. Two participants described their experience of third molar removal triggering a need for more information.

‘*. . .I had to have surgery on my gum to take a tooth out. After that I was in really bad pain and it was a struggle for me to smile or talk and laugh. So that’s why I messaged her because I thought if this is just one tiny part of my mouth, what will the rest be like.*’ (F^2^ 23)

No participants in this study reported total aversion to seeking information. Participants selectively engaged in orthognathic information sources at various stages in their decision-making journey, which ultimately led to them all feeling well informed and confident in their treatment decisions.

### Theme 2: The central role of patient-specific information from professionals and peers

Overall, verbal information specific to the patient obtained from the clinical team followed by information from similar past patients was preferred by participants. Verbal discussion allows two-way flow of information so that clinicians can determine individuals’ needs and provide relevant information. This tailored approach where patients received person-specific information was recognised as the most preferred form of information.

‘*. . .That (the consultation) would always be the priority because they’re seeing me in-person and they can give me more advice specific to me.*’ (M^7^ 27)

The joint orthognathic clinic was specifically cited as a valuable opportunity to discuss treatment with both the orthodontist and surgeon and support patients in making decisions right for them as an individual.

‘*They don’t sugar-coat it to try and get you to say yes to get you to do the operation. They just tell you the different sides, that there will be swelling 2 or 3 days after or longer, and your jaw can be sore . . . They don’t hide anything. They’re just trying to get you to make the decision for yourself . . .*’ (M^5^ 20)

Participants valued open discussions about the risks and consequences of treatment relevant to them as an individual. Conversely, some commented on a negative perception of being dismissed with a leaflet rather than being allowed to discuss matters in person.

‘*So overall, just giving you a leaflet and expecting that to be enough isn’t helpful.*’ (F^8^ 30)

When referring specifically to the British Orthodontic Society (BOS) leaflet provided by the clinical team, several patients expressed dissatisfaction describing it as too general and lacking practical utility for decision-making. A recommendation from patients to improve the usefulness of the patient information leaflets (PILs) was for diagrammatic representation to illustrate the estimated timeline of treatment rather than just written text.

‘*A timeline in it would be ideal. What to expect at each stage. . . That would be helpful so you could wrap your head around it rather than a bunch of words laid out.*’ (F^3^ 18)

Past patients also supplied patient-specific information. Speaking to patients from the same unit, of a similar age and going through a similar decision-making process, typically allayed fears of the unknown and influenced decision-making positively.

‘*. . . He had it in the same Trust as me. So it was helpful having it from the same point of view, within the same trust.*’ (F^1^ 27)

Social media provided a convenient alternative for patients to interact. Three participants used Instagram’s private message functionality to communicate with individuals who were past patients.

‘*It was just Instagram we ended up messaging I think, that was just the most convenient place that we had each other added.*’ (M^7^ 27)

Two participants used online forums, such as Reddit and The Student Room. Reddit offers a platform to pose questions to a global community of past patients and The Student Room relies on the experiences of past patients at university. The questions posed were to support their decision-making process.

‘*I just posted questions to people who had this surgery previously, like what were the benefits or do you at the time, would you have not chosen it.*’ (M^6^ 19)

Interestingly, only one participant sought to explore the views of individuals with the same malocclusion who opted not to have surgery.

### Theme 3: Internet use to supplement standard information resources

The mainstay source of information was the consultation with clinicians (maxillofacial surgeons and orthodontists). The standard supplementary information provided to patients at consultation comprises the PILs from BOS and the weblink to the BOS website, *Your Jaw Surgery*. All participants reported that these were made available. Some felt this was not enough to satisfy their information needs before making a decision and resorted to the internet for additional content.

‘*I think if I didn’t look on Instagram, I would have got a massive shock, after the surgery had happened from what I just got in clinic and the leaflets.*’ (F^2^ 23)

Patients regarded the Internet as a way to access a vast amount of up-to-date information in a timely and convenient manner.

‘*I’d always go online first because that’s just where you’ve got the biggest wealth of information.*’ (M^7^ 27)

The main concern for participants before making their decision was the fear of disliking their new facial appearance. All participants sought before and after photographs specific to their malocclusion to allay these fears and aid their decision.

‘*The results afterwards, so seeing photos of someone’s before and after, that impacts quite a bit on your decision.*’ (F^1^ 27)

Before and after photographs were perceived to be limited or missing from existing sources, leading participants to resort to online searches including Google Images, YouTube and social media.

‘*I looked at it [the BOS website], but it wasn’t something I used a lot. . . I went on YouTube and then there’s like videos of people explaining the surgery and they had slide shows of photos, of like before and after.*’ (M^6^ 19)

More specifically, inadequacies in visual aids of the immediate postoperative recovery period provided by the clinical team resulted in patients using online searches to fulfil the need for ‘real-time’ photographs of the recovery period.

‘Photos of the after results, I remember when I went for an appointment, *** [the surgeon] was telling me, after the surgery you’ll look like a chipmunk, and I saw someone walking in that I thought looked like, just after the surgery. But until I went online, I didn’t see the after surgery pictures.’ (M^6^ 19)

Social media provided the additional advantage of instant sharing offering immediate, synchronous information of an individual’s recovery.

‘*I know a lot of people have separate accounts [on Instagram]. They take pictures every week of their journey. So, I’ve looked into that quite a lot.*’ (F^2^ 23)

The Internet also served as supplementary resource when insufficient explanations during consultation prompted individuals to seek clarifications on medical terminology used.

‘*The YouTube videos were for any medical terms I didn’t understand.*’ (M^6^ 19)

Despite this, all participants reported that information from professionals was the preferred source of information. All participants used online information simultaneously with traditional sources and none used the Internet to fully replace traditional methods.

### Theme 4: Concerns over information found online

Patients trusted information from professionals online and attached value to accredited professional sites, such as the BOS online resource. Five participants demonstrated a healthy level of suspicion with regard to non-verified sources.

‘*Those websites are verified by health professionals, it’s not like Wikipedia where anyone can just edit it.*’ (M^6^ 19)

There were concerns over the reliability of the visual representation from unverified online sources such as Instagram.

‘*You don’t know what you actually looking at on Instagram. A lot can be photoshopped.*’ (F^1^ 27)

Credibility of information was evaluated by how recently it was updated, determining authorship, checking citations and cross-referencing against other sources. One individual avoided using the BOS website as she felt it was outdated.

‘*I think I just found it a bit too out of date, like the Twitter logo was wrong on it, so for me that’s just not a site I would be interested in. . .*’ (F^8^ 30)

Some participants felt they could not relate to images found online as they were too general and not applicable to them as an individual.

‘*I looked at different jaw surgeries on Instagram. . . They are really extreme, and I couldn’t relate to them. I don’t think I’ve got a prominent jaw or the opposite.*’ (F^4^ 23)

The problem of establishing applicability was further compounded by the volume and variety of surgeries and associated outcomes depicted online.

‘*. . .it was difficult to find some photos, cause it was difficult to tell what was an underbite surgery or what was an overbite surgery.*’ (M^6^ 19)

Increasing amounts of information available can lead to information overload. Patients described feelings of frustration and confusion, which in turn prolonged their decision-making.

‘*It’s easy to sometimes to go down a rabbit hole and find out stuff that isn’t necessarily important. . .*’ (M^7^ 27)

Interestingly, none of the participants reported discussing online findings and the impacts of these with the clinical team, the two activities co-existed independently, in parallel.

## Discussion

This study is the first to characterise the nuances of how patients seek information and their preferred sources during decision-making for orthognathic treatment.

In this study, all participants made informed decisions about orthognathic treatment and engaged fully with the decision-making process to undergo orthognathic surgery. This is contrary to findings by Stirling et al. (2007), that few orthognathic patients (3%) actively seek information from other sources and had not made informed decisions. This may be attributed to the change in information availability over the last two decades. For example, YouTube was founded in February 2005, only 2 years before this study was published and online information was still in its early stages of uptake. The growing accessibility of information online better enables patients to readily find a wealth of information. In addition, the elective and extended nature of orthognathic treatment, which results in noticeable facial changes, motivates patients to ensure decisions made are worthwhile.

Previously, information-seeking behaviours have been categorised into two groups: monitors or blunters (Miller, 1995). Monitors actively seek information to help them to cope with decisions and blunters actively avoid information. The findings in this study go beyond this dichotomy and identified that patients considering orthognathic treatment selectively engage with information sources through actively asking questions, avoiding certain aspects or choosing when to seek further information after a significant event. Several patients reported pre-preparing questions to ask during consultations after they had accessed information online. It suggests there may be a benefit to interventions, such as question prompt sheets as used in oncology services, which encourage patients to think about questions they want to ask (Miller and Rogers, 2018). It also highlights that information provision is not a one-time occurrence but important throughout the decision-making process. Clinicians should recognise that every patient contact is an opportunity to identify and address the evolving information needs of the individual.

Consistent with other studies, this study highlights the importance of the patient–clinician interaction in decision-making for orthognathic surgery (Broder et al., 2000; Paul et al., 2022). Negative perceptions arise when patients feel dismissed with an information leaflet or weblink rather than being allowed to discuss matters in person. Principally it is important to focus on clear verbal communication, while skilfully incorporating written and visual information into a consultation. Directly communicating to patients the purpose and key characteristics of the information aid within the consultation, rather than expecting patients to read them separately, may enhance its function as a memory aid.

Face-to-face consultation with the clinical team was the preferred source of information followed by contact with past patients as information was interactive and person-specific. This is corroborated by other studies which recognise the benefit of patients learning directly from one other (Bergkulla et al., 2017; Ryan et al., 2011). The present study found patients were establishing contact through digital social media networks as a convenient alternative to face-to-face. These routes could be used to connect patients and improve access to valuable support networks.

This study identified an unmet need for visual information specifically in the form of real-time images of postoperative recovery as well as before and after photographs directly applicable to the individual. Patients resorted to the Internet using Google, YouTube and social media to meet this need. A recent systematic review found using visual aids during decision-making, rather than relying on verbal and written information alone, results in better patient involvement in decision-making in orthodontics (Shelswell et al., 2022). Although valued, some patients had issues in establishing applicability of the images found as it was difficult to discern which malocclusion or surgeries were represented. Such decision-relevant information should be displayed with clear, explanatory captions and this would be a recommendation for professional websites in the future. Moreover, patients’ specific suggestions for incorporating diagrams into information aids align with the broader trend observed in this study, where individuals recognise the importance of visualisation of the process to aid decision-making.

Increasing amounts of information available can lead to overload, which can impede decision-making (Duran-Nelson et al., 2013). By assessing individual information requirements, the clinician is better equipped to guide patients to more relevant and valid sources. Participants reported information from the clinical team as the most trusted source of information, yet did not discuss the findings of their online information searches with the clinicians to validate it. Open discussion about online information resources during clinical consultations may encourage such information sharing. As well as providing clear verbal clarification, patient-specific concerns can be addressed and there is also an acknowledgment of the growing reliance upon online resources to gather information outside of consultations. Interestingly, in this study, none of the Internet-informed participants reported discussing online findings with their clinician; the two activities co-existed separately.

It is important to understand why patients choose certain methods over others. Kettle et al. (2017) and Flett et al. (2014) reported separately the usefulness of an online information resource and a DVD information resource through qualitative inquiries involving orthognathic patients. This study is the first, to our knowledge, that identifies the methods patients use to assess the creditability of the information used. This highlights the importance that professional websites should be kept updated with clearly stated authorship and citations.

## Limitations

The non-participation rate was 57%, so it is possible that participants who self-selected had stronger views than those who chose to not participate and which would bias the data. However, participants interviewed included a range of ages, malocclusions and genders. In addition, a diverse range of views were obtained. Nonetheless, the sample was from one orthognathic clinic under the care of the same clinical team, which may restrict transferability with regard to information provided in other units. Although thematic saturation was felt to be achieved, of course a larger sample with different socioeconomic backgrounds, ethnicities and ages may have reflected a broader range of perspectives. Further, this cross-sectional study offers individuals’ views at one time point; future research in this field would benefit from a longitudinal study from pre-decision-making to postoperative participants.

Researcher bias is the most common criticism in qualitative research (Pope and Mays, 2020). The researcher (NW), an orthodontic trainee, practised principles of reflexivity and preconceptions were minimised, where possible. The interview technique and questioning styles were conducted as recommended by Britten (1995) to minimise bias during the interviews. A second, independent researcher (NN) was involved in coding and data analysis. Transparency in data analysis and interpretation was facilitated using NVivo 12 software.

## Clinical and research implications

The findings indicate a number of implications for practice. The study gives the clinician a clearer insight into information-seeking behaviours of orthognathic patients. Clinicians have a central role in information provision and should consider the type of resource that is most useful for individual patients and ways to discuss information accessed online in consultations. This may alleviate concerns over the reliability and information overload with a positive impact on patient decision-making. Furthermore, inadequacies identified with regard to visual aids, such as real-time images of the postoperative recovery and diagrammatic representation of treatment timeline, can better inform future PIL designs.

## Conclusion

This study highlights the central role of the patient–clinician interaction in decision-making for orthognathic treatment, especially in providing information that patients value in a form that they prefer. Patients frequently access online decision-relevant information to make informed decision for orthognathic treatment. Encouraging patients to discuss online searches and their impacts with clinicians, will support shared decision-making and alleviate any concerns with the information accessed. Past patients were identified as valued sources of information and establishing contact through social media networks was found to be a convenient alternative to face-to-face contact, especially in fulfilling the identified unmet need for ‘real-time’ photographs of the recovery phase postoperatively. This study identified the need for more visual aids. Clear explanatory captions to establish applicability of photographs and diagrammatic representation of the treatment timeline are two recommendations for the design of future information aids. During the consultation, explaining the purpose and key characteristics of the information aid, rather than expecting patients to read them separately, may enhance its usefulness in decision-making.

## Supplemental Material

sj-docx-1-joo-10.1177_14653125241249494 – Supplemental material for Information-seeking behaviour in patients exploring orthognathic surgery: A qualitative study Supplemental material, sj-docx-1-joo-10.1177_14653125241249494 for Information-seeking behaviour in patients exploring orthognathic surgery: A qualitative study by Nicola Wade, Ninu Paul, Nathan Nagar, Sarah Rolland and Sarah Germain in Journal of Orthodontics

sj-pdf-2-joo-10.1177_14653125241249494 – Supplemental material for Information-seeking behaviour in patients exploring orthognathic surgery: A qualitative study Supplemental material, sj-pdf-2-joo-10.1177_14653125241249494 for Information-seeking behaviour in patients exploring orthognathic surgery: A qualitative study by Nicola Wade, Ninu Paul, Nathan Nagar, Sarah Rolland and Sarah Germain in Journal of Orthodontics

sj-pdf-3-joo-10.1177_14653125241249494 – Supplemental material for Information-seeking behaviour in patients exploring orthognathic surgery: A qualitative study Supplemental material, sj-pdf-3-joo-10.1177_14653125241249494 for Information-seeking behaviour in patients exploring orthognathic surgery: A qualitative study by Nicola Wade, Ninu Paul, Nathan Nagar, Sarah Rolland and Sarah Germain in Journal of Orthodontics
