# Supplementary material for: Information-seeking behaviour in patients exploring orthognathic surgery: A qualitative study
Source: J Orthod. 2024 May 8;52(1):63–71. doi: 10.1177/14653125241249494 (PMC11951351; doi:10.1177/14653125241249494)
Supplement: sj-docx-1-joo-10.1177_14653125241249494 – Supplemental material for Information-seeking behaviour in patients exploring orthognathic surgery: A qualitative study [file sj-docx-1-joo-10.1177_14653125241249494.docx]

| Time | Topic | Interviewer Task |
| --- | --- | --- |
| 0-5 mins | **Welcome and briefing** | Participants will be thanked for agreeing to take part in the study.  Introduce self, role in relation to the study.  Discuss the scope and purpose of the study.  Remind participants they will be recorded.  Reassure participants about confidentiality, anonymity and right to withdraw. Discuss data storage, use and sharing.  Reassure participants that part in the survey, or choosing not to, will NOT affect your future care in any way.  Describe what will happen during the interview and the format, so participants know what to expect.  Ask participants to sign the consent forms. |
| 5 mins | ***Turn on recording device*** | |
| 5-10 mins | **Warm-up/ basic questions:** | Participants will be asked:   - Each participant will be asked to introduce themselves with their chosen name and say a little about themselves.   Q. What is your chosen name (chosen name only)?  Q. What is your age?  Q. When were you first told about jaw surgery?  Q. Have you made a decision about the surgery? |
| 10-20 mins | **Why do patients seek information?** | For the following questions, the purpose of these questions is to be open and explorative and , I will not necessary to stick to the exact line of questioning or order. It is important, however, that the subject matters in the topic guides are explored and the style of questioning kept open and explorative. Not leading.  Participants will be asked:   - What information they wanted to know when deciding to have or not have jaw surgery. - The main factors that influenced their decision about whether or not to proceed with jaw surgery. - Explore any anxieties/ concerns regarding jaw surgery and braces treatment. - Where possible, discuss if COVID had any affect on their decision to have jaw surgery or not.   Q. I’m interested in understanding what information you wanted to know when deciding to have or not have jaw surgery. Could you say a bit about this?  Q. What were the most important factors that influenced your decision about whether or not to proceed with jaw surgery?  (Probe until topic exhausted) |
| 20-35 mins | **How do patients seek information?** | Participants will be asked:   - To discuss, in detail how patients sought information during decision-making. - If the stage of decision-making process influenced what information source they chose to use and what they wanted to know. - To explore information-seeking behavior fully: if they actively sought, actively avoided or passively received information and whether this behavior changed in light of any particular significant event such as COVID?   Q. Think about the process of arriving at your decision of whether or not to have jaw surgery. First describe the stages of having come to this decision, then explain what information sources (if any) you used at which stage, how did you find them (provided by care provider or actively sought)? How did you use/ access them and why did you chose this format?  Prompt: *for example verbal information from orthodontist, surgeons, friends, family, past patients, written information, audio-visual, online, social media etc.*  Q. Did you chose not to use certain information sources? Why not?  Q. How do you assess information to be credible or not?  Q. Who was involved in deciding about your jaw surgery? Who, if anyone has been most helpful? What did they do? Was anyone unhelpful? What did they do?  (Probe until topic exhausted)  Prompts:  *Q. Do you often use the same source, irrespective of the kind of information you need?* |
| 35- 40 mins | **How do patients prefer information?** | Participants will be asked:   - To discuss the preferred method in obtaining information and whether this has changed in light of any significant event such as COVID.   Q. What source/s of information do you feel is your preferred way in helping you make your decision on whether or not to proceed with surgery? Why? Has this changed in light of COVID?  Q. When do you prefer to access information in your journey? |
| 40-50 mins | **Does current information provision meet the patient’s needs during the decision-making process?** | Participants will be asked:   - To identify and evaluate any barriers to information seeking.   Q. Do you feel you had enough information to help you make a decision about having treatment? If not, why?  Q. Did you have any barriers, problems or concerns with any of the information sources used?  Q. How easy or hard was it to decide? How confident are you in your decision?  Q. Any additional information or support would you like to be provided with that would make your decision easier? Can you describe what this would look like/ comprise of?  (Probe until topic exhausted)  Prompts:  *Q. Did you have any problems in terms of accessibility, navigation, readability, poor formatting, understandability, reliability, trustworthy, not enough, too much information, concerns with the content, felt unable to ask questions, did not have enough time to read/ access information?* |
| 50-55 mins | **Closing** | Participants will be asked if they would like to add any further information they wish to add. |
| 55 mins | ***Turn off recording devices*** | |
| 55-60 mins | **Debrief session** | Thank participants for time.  Reiterate that all data is confidential, anonymous and will not be shared.  Participants will be de-briefed on the next steps of the research process.  Remind participants of contact details to get in touch by email or phone if they want to discuss any concerns or issues that arouse during the interview. |
