# Supplementary material for: Information-seeking behaviour in patients exploring orthognathic surgery: A qualitative study
Source: J Orthod. 2024 May 8;52(1):63–71. doi: 10.1177/14653125241249494 (PMC11951351; doi:10.1177/14653125241249494)

Appendix A

Information-seeking behaviour of orthognathic surgery patients: an exploratory study

Participant instructions for online interview and consent

Interview instructions

Please see instruction video below or read the instructions below:

https://www.microsoft.com/en-us/videoplayer/embed/RE3Oz24?pid=ocpVideo0-innerdiv-oneplayer&postJsllMsg=true&maskLevel=20&market=en-us

Join a Teams meeting on the web

Don't have the Teams app? You can still join a Teams meeting.

1. In your email invite, select **Join Microsoft Teams Meeting**.
2. You have two choices:
   - **Download the Windows app**: Download the Teams app.
   - **Join on the web instead**: Join a Teams meeting on the web. Please use Google Chrome and not Safari*.
3. Type in your name, please use your chosen name.
4. Choose the audio and video settings you want. Please unmute and leave the video for purposes on the research interview.
5. Select **Join now**.
6. Depending on meeting settings, you'll get in right away, or go to a lobby where someone in the meeting can admit you

- Supported Web browsers to use: Google Chrome, the latest Microsoft Edge version (plus last two versions)
- Unsupported Web browser NOT to use: Safari, Firefox, Internet Explorer 11, Microsoft Edge version RS2 or later.
- If required, Google Chrome can be downloaded/ installed for free from https://www.google.com/intl/en_uk/chrome/

Join a Teams meeting from the app

1. From you **Calendar**, select **Join** on an in-progress meeting.
2. Choose the audio and video settings you want.
3. Select **Join now**.

Information-seeking behaviour of orthognathic surgery patients: an exploratory study

Consent instructions

1. In your email invite, select **Review and sign**.

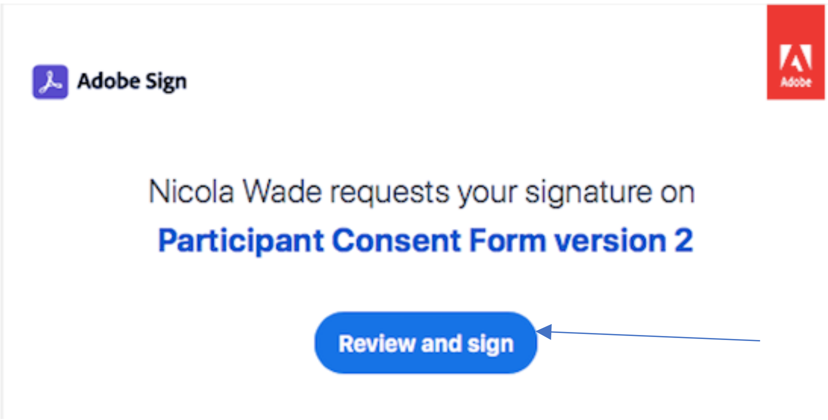

2. This opens the form in a web browser (preferably Chrome, not Safari).
3. **Review the form and sign** where indicated. You can choose to draw, type or use an image for your signature. Then click Apply.

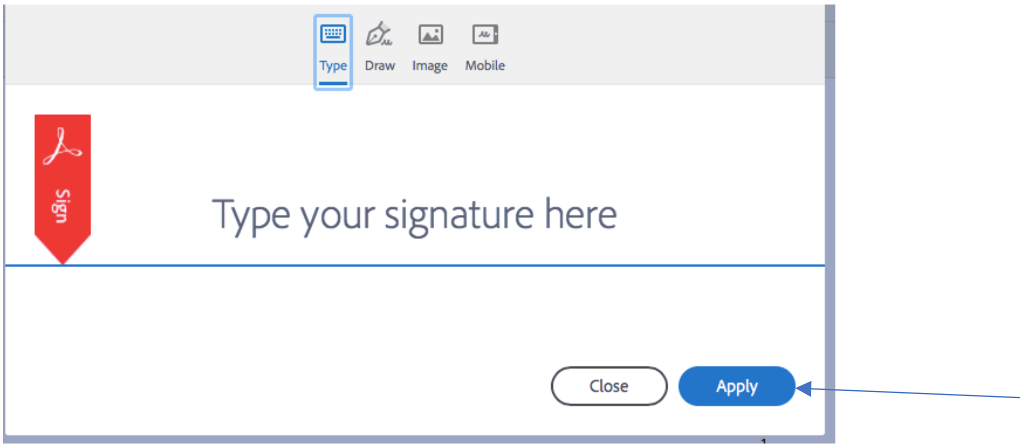

4. Send your form, this will send your completed form to yourself and the research team, to be stored securely and confidentially.

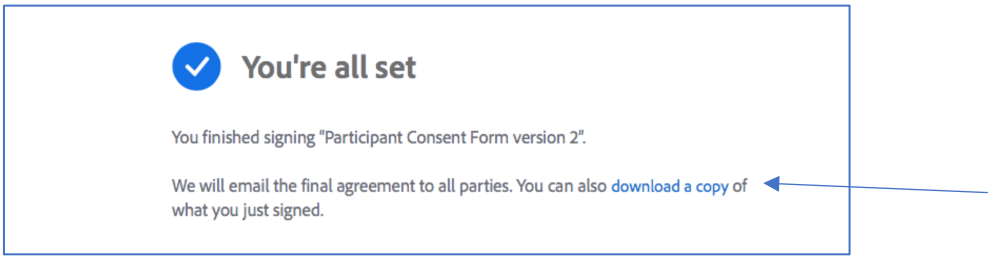

Supplement: sj-pdf-2-joo-10.1177_14653125241249494 – Supplemental material for Information-seeking behaviour in patients exploring orthognathic surgery: A qualitative study [file sj-pdf-2-joo-10.1177_14653125241249494.pdf]
